# The nonlinear cysteine redox dynamics in the *i*-space: A proteoform-centric theory of redox regulation

**DOI:** 10.1016/j.redox.2025.103523

**Published:** 2025-02-05

**Authors:** James N. Cobley, Panagiotis N. Chatzinikolaou, Cameron A. Schmidt

**Affiliations:** a The University of Dundee, Dundee, Scotland, UK; b Aristotle University of Thessaloniki, Serres, Greece; c Cells and Systems Biology lab, ECU biology, USA

**Keywords:** Oxiforms, Redox regulation, *I*-space, Nonlinear, Cysteine proteoforms

## Abstract

The post-translational redox regulation of protein function by cysteine oxidation controls diverse biological processes, from cell division to death. However, most current site-centric paradigms fail to capture the nonlinear and emergent nature of redox regulation in proteins with multiple cysteines. Here, we present a proteoform-centric theory of redox regulation grounded in the *i*-space. The *i*-space encapsulates the theoretical landscape of all possible cysteine proteoforms. Using computational approaches, we quantify the vast size of the abstract *i*-space, revealing its scale-free architecture—elucidating the disproportionate influence of cysteine-rich proteins. We define mathematical rules governing cysteine proteoform dynamics. Their dynamics are inherently nonlinear, context-dependent, and fundamentally constrained by protein copy numbers. Monte Carlo simulations of the human protein PTP1B reveal extensive *i*-space sampling beyond site-centric models, supporting the “oxiform conjecture”. This conjecture posits that highly oxidised proteoforms, molecules bearing multiple oxidised cysteines, are central to redox regulation. In support, even 90%-reduced proteomes can house vast numbers of unique, potentially functioanlly diverse, oxiforms. This framework offers a transformative lens for understanding the redox biology of proteoforms.

## 1 Introduction

The sulfur atom in the amino acid cysteine profoundly influences protein structure and function [1], supporting fundamental redox reactions, including metabolising reactive oxygen species (ROS), guiding protein folding, and hosting essential cofactors [[2], [3], [4], [5], [6]]. Most current paradigms for understanding cysteine's role in redox regulation and oxidative stress use “site-centric” models. In these models, a given function (*f*) is explained by residue-specific transitions from one sulfur-specific chemical form—chemotype [7,8]—into another [[9], [10], [11], [12], [13], [14], [15], [16], [17], [18]].

For example, ROS inactivate the tyrosine phosphatase activity of PTP1B by oxidising the active-site cysteine (Cys215) from a deprotonated thiolate (sulfur = −2) to a sulfenic acid (sulfur = +1) in redox phase space [[19], [20], [21], [22], [23]]. This vast redox phase space can be simplified by letting the -2-sulfur state be reduced (0) and collapsing all other states that are biological encountered (i.e., −2, −1, 0, +1, +2, +4, +6) to oxidised (1); where oxidised includes many chemotypes, from sulfenic acids to mixed disulfides [24]. In binary (reduced = 0, oxidised = 1) redox phase space, PTP1B inactivation can be described as a linear operation with the transition mapping to a line connecting point *A* (Cys215-0) to point *B* (Cys215-1). Hence, for PTP1B, *f* could simply be described as *B* (Cys215-1).

However, this linear model cannot account for the emergent properties resulting from interactions between multiple cysteines. Such interactions demand a nonlinear proteoform-centric paradigm to capture the hierarchical and dependent transitions in multi-cysteine proteins. Kelleher and colleagues [25] coined the term proteoform to “*designate all of the molecular forms in which the product of a single gene can be found*”. They formalised the idea that one protein can produce multitudes of biochemically unique species with distinct structural and functional properties via genetic mutations, splice variants, and post-translational modifications (PTMs) [26,27]. Cysteine redox proteoforms (*i*) can be conceived as an *i*-space housing all of the theoretically possible cysteine residue- and redox phase space-defined forms that a protein molecule can adopt [28].

In the proteoform paradigm [29], linear transitions involving single residues on proteins that possess multiple cysteines are unlikely to fully explain redox regulation or oxidative stress [30]. Rather, these phenomena might be more fully explained by the nonlinear dynamics of proteoforms. In the case of PTP1B, *f* cannot simply be described as *B* when *B* just considers Cys215-oxidised. Instead, the redox phase space of multiple cysteines must be accounted for to describe *B*. While *B* could simply map to 0001000000 (Cys215 = site 4), *f* might involve other proteoforms, such as 0101000100. In line with the redox code [31,32], redox regulation may involve cysteine oxidation dependencies and hierarchies. For example, 0101000100 modulates *f* by sustaining it over time when position 2 and 8 are reduced before 4. Hierarchical dependencies demonstrate how the emergent complexity of proteoforms can control redox regulation.

Current progress is rate-limited by the challenges of measuring cysteine proteoforms [33], including the sensitivity and resolution issues that limit their detection [30,34]; and the lack of a formal proteoform-centric theoretical framework [28,30]. To elaborate a formal framework, we used computational methods to (a) compute the size of the human theoretical *i*-space, (b) calculate the biologically accessible *i*-space, (c) map *i*-states to percentage redox grades, (d) define the rules governing proteoform dynamics, and (e) model these dynamics. This work defines a proteoform-centric theory of redox regulation.

## 2 Results

### 2.1 Mathematically formalising the theoretical *i*-space

Let us begin by defining the terms of a proteoform-centric formalism.

• An ***i*-state** refers to a unique cysteine proteoform in a given theoretical *i*-space.
• A given theoretical ***i*-space** houses all of the possible *i*-states in a given redox phase space. The “theoretical *i*-space” is abstract, defining what can hypothetically exist (not what does exist) in a given biological system, from an organism to a single cell.
• A given **redox phase space** refers to the number of dimensions that are operated to calculate the *i*-space. For example, a **binary** redox phase space.
• **Binary redox phase space** means that the reduced state is denoted as 0 whereas the oxidised state, regardless of what specific chemotype it is from sulfenic to sulfonic acids, is denoted as 1. As a result, it can be described as 2-dimensional in the *n*, see equation (1), basis. Hence, *n*-2-dimensional. A higher *n*-X-dimensional, where X is an integer greater than 2, can be used to describe as many distinct chemotypes as desired. For example, an *n*-4-dimensional space could describe reduced (0), sulfenic acid (1), sulfinic acid (2), and sulfonic acid (3).
• A **biologically accessible *i*-space** defines the maximum number of specific *i*-states that can exist in a given system at a single point in time. By definition, the limit can never exceed the theoretical *i*-space. The maximum number of specific *i*-state that can exist is independent of the *n*-integer used to calculate the theoretical *i*-space. This is because every *i*-state state regardless of what it is can be described in the binary reduced or oxidised basis.

Formally, a protein molecule containing *R*, where *R* is an integer, cysteine residues can be reduced (0) or oxidised (1) in *n*-2-dimensional redox phase space. In the simplest possible case, a monothiol (*R* = 1) can hypothetically exist in the 0 or 1 *i*-states, yielding a theoretical *i*-space of 2. Hence, the theoretical *i*-space for a given protein molecule can be calculated using equation (1).

(1) $$Protein\;molecule\_specific\;theorerical\;i\_space=n^{R}$$

Where.

• *n* is the redox phase space dimension integer.
• *R* is the cysteine residue integer.

Equation (1) can be extended to calculate the theoretical *i*-space for a given species by summing the *i*-space of each protein in a reference proteome. Each protein is treated as a single copy (ignoring duplicates and complexes), and its *i*-space is calculated individually using its *R* value.

(2) $$Species\_specific\;theoretical\;i\_space=\sum_{k=1}^{M}n^{R_{k}}$$

Where.

• *n* is the dimensional redox phase space integer (e.g., 2 in binary)
• *R*_*k*_ is the number of cysteine residues in the *k*-th entry in the proteome.
• *M* is the number of protein entries in the proteome (e.g., 20,434 in humans).

In sum, equations (1), (2)) formalised the theoretical *i*-space concept.

### 2.2 The size of the theoretical human *i*-space is vast

**Fig. 1:**
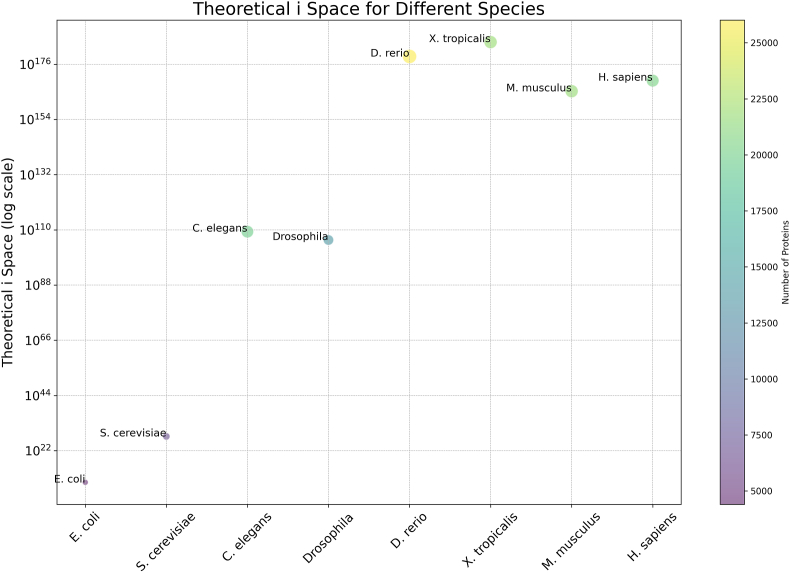
Theoretical *i*-space for Different Species. The bubble plot illustrates the theoretical *i-*space across various species. The y-axis represents the theoretical *i-*space on a log scale, while the x-axis lists the species. Bubble sizes correspond to the number of proteins in each species, and the colour gradient reflects the same. To execute the code for plotting the bubbles on the graph, two extreme outliers were omitted: M9PB30 with 2647 cysteine residues in *Drosophila* and A0A8M9P2Z8 with 1124 cysteine residues in *D. rerio*.

Having formalised the mathematics, we quantified the size of the theoretical *i*-space in humans. Solving equation (2) using the reference human proteome revealed a theoretical limit of 3.02 x 10^169^ *i*-states in humans in binary redox phase space (supplementary data file 1–2). The sheer size of this number demonstrated that the hypothetical potential to diversify the cysteine proteome by forming unique *i*-states from a finite set of protein molecules—19,814 cysteine containing proteins with 262,025 cysteine residues—is vast. For context, many model species possessed vast theoretical *i-*spaces (Fig. 1, Table 1, & supplementary data file 3), indicating that this hypothetical potential for diversifying the proteome by forming unique *i*-states is not unusual.

**Table 1:** Quantitative information on the selected proteome parameters, such as the number of proteins, by selected model species. Cysteine residue mean, median, and mode values are reported as integers or rounded to the nearest integer to reflect the nature of cysteines (i.e., a half cysteine is biologically implausible). The data were extracted by analysing their reference proteomes per the methods section.

	E. coli	S. cerevisiae	C. elegans	Drosophila	D. rerio	X. tropicalis	M. musculus	H. sapiens
***Proteins***	4404	6735	19,832	13,824	26,015	21,841	21,709	20,434
***Amino acids***	1354446	3026653	8135557	7402372	15185681	12515240	11747732	11409411
***Cysteine residues***	15,758	39,917	168,776	142,220	362,213	302,596	277,381	262,025
***Cysteine (%)***	1.2	1.3	2.1	1.9	2.1	2.4	2.4	2.3
***Cysteine proteins***	3701	6106	18,709	13,041	25,332	21,265	21,014	19,817
***Cysteine proteins (%)***	84.0	91.7	93.6	94.3	97.4	97.4	97.2	97.0
***Mean***	4	6	8	10	14	14	13	13
***Median***	3	4	6	7	10	10	9	9
***Mode***	1	4	1	2	4	6	6	3
***Classes***	25	47	135	122	196	196	172	174
***Range***	0–31	0–92	0–363	0-2467	0-1124	0–613	0–549	0–563
***i-mode (n)***	2 (669)	16 (729)	2 (1520	4 (983)	4 (1534)	64 (1378)	64 (1316)	8 (1298)
***i-space***	2.5 x 10^9^	4.95 x 10^27^	1.9 x 10^109^	6.7 x 10^796^	2.27 x 10^338^	6.79 x 10^184^	1.84 x 10^165^	3.02 x 10^169^

**Fig. 2:**
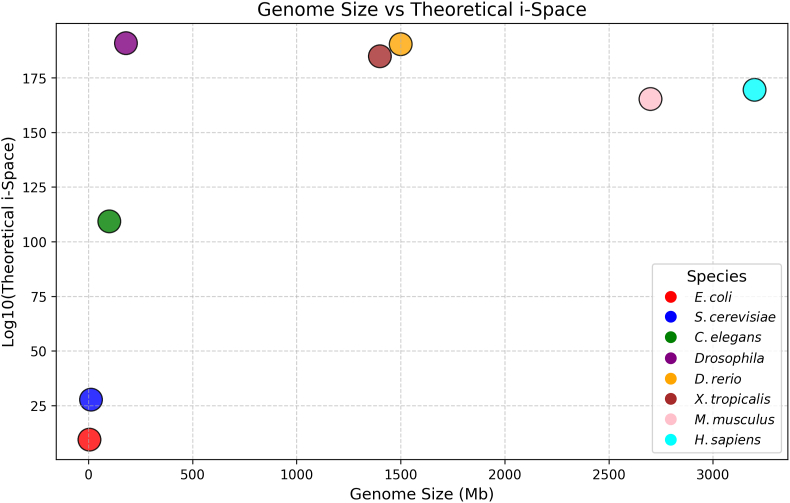
Scatter plot of the Theoretical *i*-space for Different Species against their genome size. The y-axis represents the theoretical *i-*space on a log scale, while the x-axis lists the genome size in megabase pairs (Mb). To execute the code for plotting the bubbles on the graph, two extreme outliers were omitted: M9PB30 with 2647 cysteine residues in *Drosophila* and A0A8M9P2Z8 with 1124 cysteine residues in *D. rerio*.

When plotted against genome size (Fig. 2), the abstract theoretical *i*-space, like the number of proteins, does not account for the complexity of the species, the so-called C-value paradox [35]. Perhaps, the biologically accessed *i*-space correlates with the perceived complexity of the species.

This potential to diversify the proteome at the PTM-level applied to every amino acid. To calculate the theoretical PTM-space, we modified equation (2) and operated an *n*-2-dimensional (unmodified or modified) phase space for each amino acid. The *n*-2 phase space for each amino acid is justified. Even glycine with a simple side chain—one hydrogen atom—can exist in multiple PTM-states, such as the glycl free radical state [36]. We estimated that 10^10340.380^ proteoforms are theoretically possible in human PTM-space, demonstrating the vast potential for proteome speciation at the PTM-level.

While every amino acid had a large PTM-space, their contribution to the total space varied as a function of their frequency. As only methionine and tryptophan are used less frequently than cysteine [37], the theoretical *i*-space 3.02 x 10^161^ represented a miniscule share of the overall binary PTM-space. However, the *n*-2-dimensional phase space does not account for the fact that some amino acids, especially cysteine [38], can exist in multiple different PTM states [39]. A revised script that used higher-dimensional *n* values, estimated a theoretical PTM-space of 10^5826598.665^ proteoforms. Only lysine and serine contributed more to this space than cysteine—10^445172.615^ proteoforms—due to their higher frequency and expanded phase space vs. other amino acids. The PTM-space of lysine and cysteine also crosstalk due to the potential for these two residues to be crosslinked [40]. Hence, cysteine residues are a major potential source of proteoform speciation in humans.

In sum, equation (2) set an upper limit of 3.02 x 10^169^ and 10^445172.615^ unique human cysteine proteoforms when the redox phase space integer was set to 2 and 50, respectively. These numbers form part of a vast PTM-space comprising 10^5826598.665^ proteoforms.

### 2.3 The theoretical *i*-space is defined by a scale-free network architecture

**Fig. 3:**
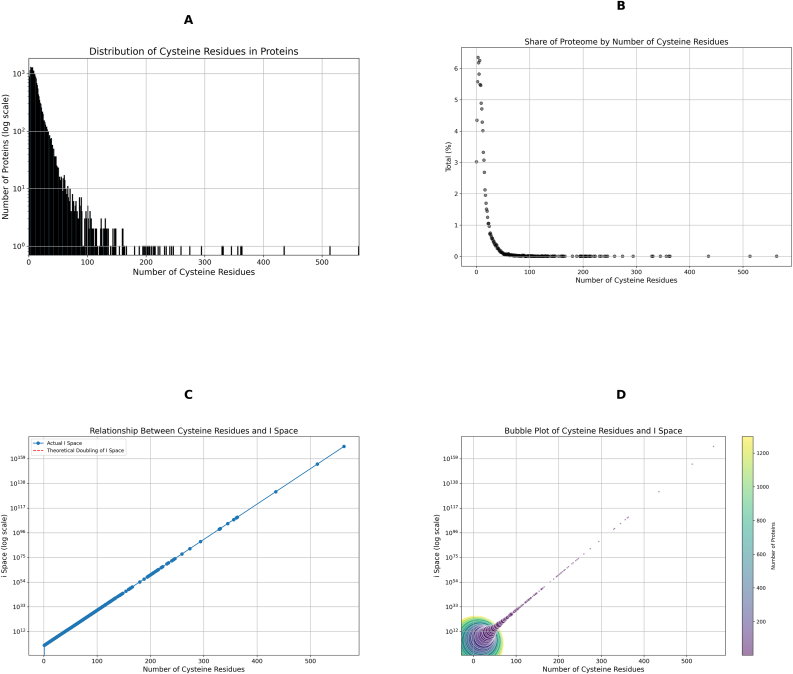
Distribution and Theoretical *i*-Space Analysis of Cysteine Residues in the Human Proteome. (A) Histogram displaying the distribution of cysteine residues across proteins in the reference human proteome, revealing a marked leftward shift, with most proteins containing fewer than 20 cysteine residues. The long tail reflects relatively rare proteins with more than 100 cysteine residues. (B) Share of Proteome by Number of Cysteine Residues illustrating the inverse relationship between the number of cysteine residues and the percentage share of the proteome. Proteins with a high number of cysteine residues have a lower percentage share of the proteome, while those with fewer cysteine residues account for a larger share. (C) Relationship Between Cysteine Residues and *i*-Space. The plot demonstrates the theoretical doubling of the *i*-space with increasing cysteine residues. The linear relationship on a log scale confirms that the *i*-space doubles with each additional cysteine residue. (D) Bubble Plot of Cysteine Residues and *i*-Space. This bubble plot visualises the relationship between cysteine residues and the *i*-space, with the bubble size representing the number of proteins. The x-axis shows the number of cysteine residues, and the y-axis (log scale) indicates the *i-*space. The colour gradient, from purple to yellow, corresponds to the number of proteins, highlighting that a small number of proteins with high cysteine counts contribute disproportionately to the total *i*-space.

Next, we explored the theoretical *i*-space as a function of its composition—considering discrete single copies of each protein unit—and its nature on an abstract mathematical plane. As the protein molecule-specific theoretical *i*-space is mathematically related to *R* [28], we plotted cysteine residue distribution frequencies. They were unevenly distributed across the 2-log *R* range that spanned 0 to 563 cysteine residues. Most proteins had less than 20 cysteines, resulting in a pronounced leftward shift in the cysteine distribution histogram (Fig. 3A). Frequent low integer *R* values confirmed that for most proteins the theoretical *i*-space is finite (e.g., *i* = 8 for the 1298 proteins in the *R* = 3 series). Hence, the share of each *R* series to the overall percentage of proteins in the proteome decreased as an inverse function of *R* (Fig. 3B).

To explore the inverse function, we stratified the cysteine residue distribution ranges using 10, 20, 50, and 100 as the cut-off classifiers for 1–100, 101–200, 200–300, and >301 cysteine residues, respectively (Table 2). Most of the human cysteine proteome (57.8 %) contained between 1 and 10 cysteine residues (23.3 % of the total). The 26.9 % of proteins with 11–20 cysteine residues accounted for 29.4 % of all the cysteine residues. Combined the 1–20 cysteine residue ranges represented 83.7 % and 52.7 % of the proteins and cysteine residues in the human cysteine proteome, respectively. The theoretical *i*-space for >80 % of the human proteome is 6.83 x 10^8^. Only 105 proteins (0.052 % of the proteome) possessed >100 cysteine residues. Of these, 27 proteins had >200 cysteine residues. Hence, the theoretical *i-*space for 99.95 % of the human proteome is 1.76 x 10^31^ *i*-states.

**Table 2:** Reference values by cysteine residue classifier range in the human proteome. The reference values cover the number of proteins and cysteine residues in each range, their percentages of the overall total, and the *i*-space for each range.

Classifier range	Proteins	Proteins (%)	Cysteine residues	Cysteine residues (%)	*i-space*
1–10	11254	61113	61113	2.08E+06	2.08E+06
11–20	5334	77040	77040	6.81E+08	6.81E+08
21–30	1683	41657	41657	2.64E+11	2.64E+11
31–40	784	27388	27388	1.32E+14	1.32E+14
41–50	328	14670	14670	5.33E+16	5.33E+16
51–60	135	7494	7494	3.42E+19	3.42E+19
61–70	84	5472	5472	1.89E+22	1.89E+22
71–80	48	3633	3633	1.26E+25	1.26E+25
81–90	41	3534	3534	1.20E+28	1.20E+28
91–100	21	2036	2036	9.66E+30	9.66E+30
101–120	29	3129	3129	1.69E+36	1.69E+36
121–140	26	3338	3338	8.44E+41	8.44E+41
141–160	13	1953	1953	4.41E+48	4.41E+48
161–180	4	671	671	1.53E+54	1.53E+54
181–200	6	1171	1171	2.18E+60	2.18E+60
201–250	15	3303	3303	1.29E+74	1.29E+74
251–300	3	827	827	3.18E+88	3.18E+88
301–400	6	2085	2085	2.36E+109	2.36E+109
401–500	1	435	435	8.87E+130	8.87E+130
501–600	2	1076	1076	3.02E+169	3.02E+169

The perfect linear relationship between the *i*-space and the cysteine residue integer (R^2^ value = 1), where the *i*-space doubled each and every time the number of residues increased by 1, unmasked the disproportionate influence of a few proteins on the theoretical *i*-space (Fig. 3C and D). One protein accounted for essentially the entire 99.9 % recurring *i*-space. With 563 cysteine residues, spondin can theoretically form 3.02 x 10^169^ *i*-states. A number so large that the notational value stayed at 3.02 x 10^169^ when all the other *i*-states were added to it because of the 15-log difference between the *i*-spaces for spondin and titin (*R* = 513). One must subtract the 3 proteins with the most cysteine residues (spondin, titin, & IgGFc-binding protein, *R* = 435) before the summed total for the remaining 19,813 proteins changes from the notational one for fibrillin-2 (*R* = 363):

**The *i*-space for 19,813 proteins = 2.36 x 10**^**109**^. [Fibrillin-2 (1.88 x 10^109^) + all other protein-specific *i*-states]

The disproportionate influence of a few proteins with large *R* integers on the theoretical *i*-space suggested a scale-free network [40], obeying the power-distribution laws evident in the mathematics of equation (1). In this regard, most protein nodes in the cysteine proteome network have 1–10 cysteine residues. Even though the few hubs with many residues were rare they explained most of the theoretical *i*-space.

As an abstract mathematical plane, the *i*-space is not intended to be a direct map of a biological redox network, as reviewed by Dietz and Hell [41]. While a biological redox network will be a function of evolving ensemble of *i*-states drawn from the theoretical *i*-space, the contribution of a single copy of a protein molecule to this abstract space does not, and is not intended, to correspond to its biological importance. For example, thioredoxin 1, with 5 cysteines contributes just 32 *i*-states to the abstract *i*-space, yet it is a critical redox hub [[42], [43], [44]]. A central message of the present work is that protein function and regulation often involve a broader sampling of the theoretical *i*-space than previously recognised.

In sum, the abstract *i*-space exhibited a scale-free network architecture due to the power-based mathematics of equation (1).

### 2.4 A mathematical framework for calculating the biologically accessible *i*-space

While any *i*-state housed in the theoretical *i*-space can hypothetically form over time, a mathematical constraint must cap the number of unique proteoforms that can form at any one point in time in a given biological reference frame. It “must” be limited because the estimated number of molecules in the “average” human body (7 x 10^27^) is 134-logs smaller than the theoretical *i*-space.

Logically, the presence of specific proteins may dictate the theoretical *i*-space in a given biological system. For example, the lack of p53 would decrease the theoretical *i*-space by 1024 proteoforms. Hence, the biologically accessible *i*-space is influenced by the protein expression argument codified in equation (3).

(3) $$\sum_{k=1}^{E}n^{R_{K}}$$

Where.

• *n* is the dimensional redox phase space integer.
• *R*_*k*_ is the number of cysteine residues in the *k*-th entry in *E.*
• *E* is the number of expressed protein entries, one copy per entry, in the given system.

To implement equation (3), we analysed a proteomic HeLa cell dataset [45] using a script for matching proteins to their UniProt accessions. The 9906 matched proteins (supplementary data file 4) and their 128,541 cysteine residues represent 48.5 % and 49.1 % of the human proteome and cysteine proteome, respectively. Fewer cysteine residues classes (−38 classes) and proteins (−50 %) concomitantly limited the *i*-space per class vs. the reference human proteome. For example, the theoretical *i*-space limit for the 1–20 residue range contracted by 50.6 % from 6.84 x 10^8^ in the reference proteome to 3.46 x 10^8^ in HeLa cells (Table 3). Equation (4) constrained the theoretical *i*-space by 15-logs from 3.02 x 10^169^ to 2.68 x 10^154^ in HeLa cells vs. the reference human proteome due to the absence of spondin.

**Table 3:** The theoretical *i*-space limit for each cysteine residue series between 1 and 20 in the reference human proteome and a published HeLa cell proteome. Less proteins in the HeLa proteome vs. the reference decrease the theoretical *i-*space in each series on expression grounds per equation (4).

	Reference		HeLa		
Cysteine residue class	Proteins	*i-*space	Proteins	*i-*space	HeLa vs. ref *i-*space (% decrease)
**1**	888	1.78E+03	504	1.01E+03	**−56.76**
**2**	1140	4.56E+03	566	2.26E+03	**−49.65**
**3**	1298	1.04E+04	606	4.85E+03	**−46.69**
**4**	1263	2.02E+04	607	9.71E+03	**−48.06**
**5**	1189	3.80E+04	567	1.81E+04	**−47.69**
**6**	1278	8.18E+04	618	3.96E+04	**−48.36**
**7**	1121	1.43E+05	532	6.81E+04	**−47.46**
**8**	1116	2.86E+05	578	1.48E+05	**−51.79**
**9**	999	5.11E+05	446	2.28E+05	**−44.64**
**10**	962	9.85E+05	404	4.14E+05	**−42.00**
**11**	876	1.79E+06	378	7.74E+05	**−43.15**
**12**	820	3.36E+06	361	1.48E+06	**−44.02**
**13**	679	5.56E+06	317	2.60E+06	**−46.69**
**14**	628	1.03E+07	270	4.42E+06	**−42.99**
**15**	548	1.80E+07	233	7.63E+06	**−42.52**
**16**	434	2.84E+07	207	1.36E+07	**−47.70**
**17**	399	5.23E+07	207	2.71E+07	**−51.88**
**18**	347	9.10E+07	164	4.30E+07	**−47.26**
**19**	308	1.61E+08	154	8.07E+07	**−50.00**
**20**	295	3.09E+08	156	1.64E+08	**−52.88**

When it is expressed, protein concentration may determine the biologically accessible *i*-space. Even an unrealistically high concentration of 1 mol of spondin, would contract *i*-space by 146-logs from 3.02 x 10^169^ to 6.022 x 10^23^. Hence, the biologically accessible *i*-space may be limited by a protein concentration argument as codified in equation (4).

(4) $$\sum_{k=1}^{E}\min\left({i\_space}_{k},N_{k}\right)$$

Where.

• *i*-space_*k*_ is the *i*-state integer for the *k*-th protein entry.
• *N*_*k*_ is the copy number for the *k*-th protein entry.
• *E* is the number of expressed protein entries in the given system
• min(*i*-space_*k*_, *N*_*k*_) is the minimum value between *i*-space_*k*_ and *N*_*k*_*.*
• The summation is over all protein entries *k* = 1 to *E.*

To implement equation (4), we retrieved an *R* value from the reference human proteome for the 8099 proteins with estimated protein copy numbers (*N*) per HeLa cell [45] (supplementary data file 5). The summed *N* value totalled 2.05 x 10^9^ protein molecules per HeLa cell. Compared to equation (3), equation (4) constrained the theoretical *i*-space by 147-logs from a maximum of 2.68 x 10^154^ to 4.04 x 10^7^ unique *i*-states per HeLa cell.

Higher dimensional *n*-space are similarly constrained. Using the above-mentioned dataset, one will arrive at 4.04 x 10^7^ unique *i*-states regardless of whether one started with an *n*-2 or *n*-50-dimensional redox phase space derived *i*-space. The only difference is the magnitude of the log-scale reductions from the theoretical to the biologically accessible *i*-space. Even if the theoretical *i*-space is 10^445172.615^, to include all known chemotypes, the biologically accessed *i*-space remains 4.04 x 10^7^ per HeLa cell.

In sum, the vast majority theoretical *i*-space housing all of the possible unique cysteine proteoforms cannot actually be biologically accessed at a single point in time in a given system. While it is a limitation of this work that they were only applied to one dataset, the protein expression and concentration arguments codified in equations (3), (4)) define a universal mathematical framework for calculating the biological accessible *i*-space of a given system.

### 2.5 A 90%-reduced proteome can support 10^5^ unique oxiforms at a single point in time

To estimate how many oxiforms, proteins with at least one oxidised cysteine residue [28], might form in a HeLa cell at an empirically-grounded cysteine proteome redox state of 10%-oxidised [46], we defined the percentage redox grade increments (*%*_*g*_) for each protein per equation (5):

(5) $${\%}_{g}=\frac{R}{100}$$

Then, we determined the number of these percentage grades (*N*_*g*_) using equation (6):

(6) $$N_{g}=R+1$$

For example, solving equations (5), (6)) for the *R* = *3* series, yielded 4 grades with oxidation states of 0 %, 33.3 %, 66.6 %, and 100 %.

To distribute the *i*-space across the grades, we represented each *i*-state as a binary string, where each digit (0 or 1) indicates whether a given cysteine residue is reduced (0) or oxidised (1). The binary strings were then grouped into grades based on the total number of 1's they contained, which corresponds to the number of oxidised cysteines. This intuitive method enabled us to map the ensemble of binary strings to the correct redox grade, as illustrated for the *R* = 3 series.

0 % = 000
33.3 % = 100, 010, 001.
66.6 % = 110, 011, 101.
100 % = 111
**Sum**: 1 + 3 + 3 + 1 = 8.

These steps enabled us to distribute the *N* values in supplementary data file 5 by their percentage redox grade and calculate the minimum number of unique cysteine proteoforms at *P* = 0.1 (10%-oxidised). While every accessed grade gives at least one unique proteoform, the maximum for each cysteine oxidation integer is set by the number of theoretically possible proteoforms for that grade. Even when there are 215 *R* = 3 molecules in the 33.3%-oxidised grade, only unique proteoforms—100,010,001—will be counted. Duplicates are discarded. A 10%-oxidised redox state corresponded to a minimum and maximum of 1.05 x 10^5^ and 9.76 x 10^6^ unique proteoforms, respectively (supplementary data file 6). Subtracting %-oxidised states, yielded a maximum of 9.75 x 10^6^ oxiforms per HeLa cell.

**Fig. 4:**
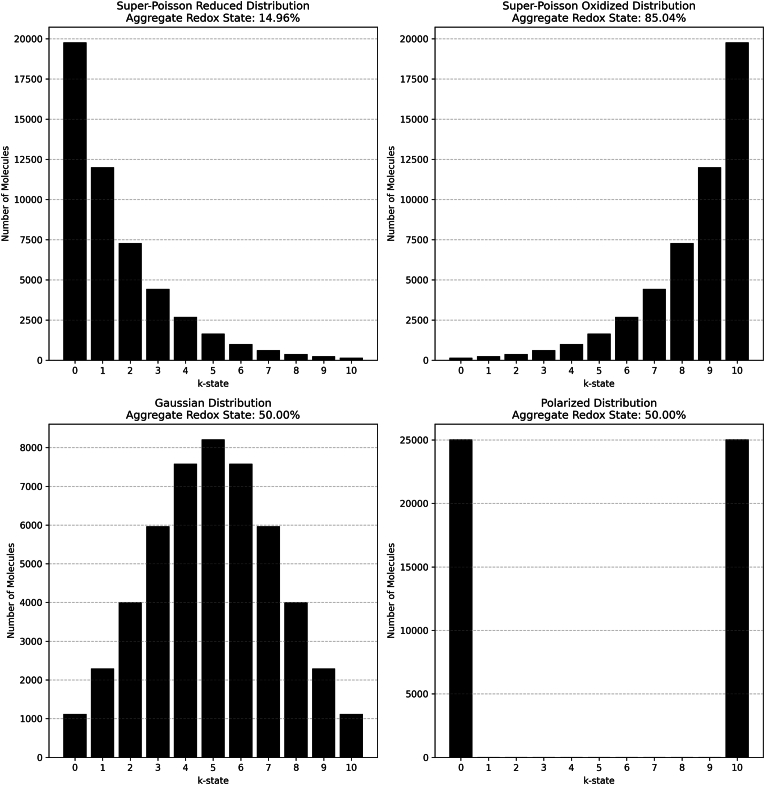
Illustrative examples of a population of 50,000 *R* = 10 molecules assigned in by cysteine oxidation integer according to different distribution schemes. Note how the Gaussian and bimodal schemes create a radically different sampling of the number of oxidised cysteines while yielding the same aggregated redox state. The impact on the sampled *i*-space is marked, all 1024 proteoforms could be sampled in the Gaussian scheme whereas just 2 would be in a bimodal scheme. Fig. 4 was automatically generated using the Histogram_plot_r10.py script.

The above-mentioned code set every protein entry into the 10%-oxidised state, enforcing homogeneity. In reality, the distribution of populations of protein molecules will vary. To illustrate this concept (Fig. 4), we plotted 50,000 copies of a molecule in the *R* = 10 series into super-Poisson reduced, super-Poisson oxidised, Gaussian, or Polarised (bimodal) distribution schemes. These distribution profiles represented an attempt to model the potential for proteins to reside in different redox environments.

To illustrate this concept, we allocated the 3000 most abundant proteins to a super-Poisson reduced distribution to maintain a highly-reducing state without fixing it to 10%-oxidised, capturing the potential for single cell heterogeneity [47]. The remaining proteins were randomly assigned to a super-Poisson reduced, super-Poisson oxidised, Gaussian, Random, or Polarised (bimodal) distribution. The resultant 16.8%-oxidised proteome redox state can support a minimum and maximum of 9.03 x 10^4^ and 7.16 x 10^6^ *i*-states, respectively (supplementary data file 7).

Setting the top 100 most abundant proteins to 0-oxidised and distributing the rest in a super-Poisson reduced, yielded an 8.5%-oxidised proteome redox state. This redox state could support 1.05 x 10^5^ and 3.19 x 10^6^ unique cysteine redox proteoforms, respectively (supplementary data file 8). Despite a 50%-decrease in cysteine oxidation, the minimum number increased because of the high molecule counts in low percent redox grades, enabling a high sampling of the minimum value at high *R* integers. Hence, even a slightly oxidised proteome can theoretically house many unique *i*-states.

**Fig. 5:**
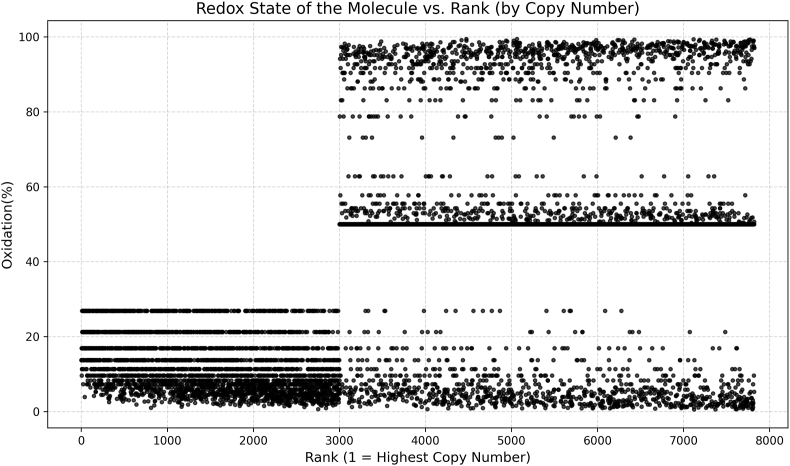
Cysteine oxidation by protein copy number rank plot for each protein entry. This plot was automatically generated from the supplementary data file 8 using the script Rank_OX_plot.py to illustrate two points. First, abundant molecules in the reduced state weight the overall redox state of the cell towards the reduced state even when there can be many molecules in higher-order *k*-states. Second and related, there can be extreme heterogeneity in these redox states. It can also be seen that there are heavily and sparsely populated percentage oxidation zones irrespective of rank.

The vast number of ways the molecules and their redox grades can be combined to give empirical redox states mean that many permutations are possible. Relatedly, the most abundant molecules have an outsized impact on the total redox state (Fig. 5). However, they tended to have a low theoretical *i*-space. For example, the 1000 most abundant proteins accounted for 85 % (1.70 x 10^9^) of the total *N*, but less than 1 % of the theoretical *i*-space. Moreover, these sampling scripts can be revised to take into account proteins known to be susceptible to oxidation using resources, such as OxiMouse and CysDB [48,49].

The sampled *i*-space can be expanded in two important ways. First, at any given point in time, there will be heterogeneity in the sampled *i*-space sets, where each set is the sampled *i*-space per cell, across the cells comprising a given system. The sampled *i*-space for the system, the system set, will inevitably be greater than any single set due to the unique nature of each individual set. The probability of two individual sets being identical is vanishingly low, which means the set of biologically accessed *i*-states expands when each cell is merged to produce a tissue ensemble. Second, the sampled *i*-space will be expanded across time, as the *i*-state of each individual set evolves. Given 3.7 x 10^13^ cells evolve over many decades [50], it is possible that a substantial fraction of the theoretical *i*-space might be biologically accessed over a typical human lifespan [28].

In sum, even highly-reduced proteomes can still theoretically house many unique oxiforms, supporting the idea that a large fraction of the biologically accessible *i*-space may be sampled. This sampling can occur in an effective infinite number of ways due to the vast degrees of freedom that are available to billions of protein molecules. Put differently, there are many mathematical solutions to a given percentage cysteine redox state.

### 2.6 A law: the number of proteoforms always equals the number of protein molecules

A law: The number of proteoforms (*P*) in a given biological reference frame will always equal the number of protein molecules (*N*) in the frame (equation (7)). This is because every protein molecule is a proteoform [51]. The infinite *P*-space arising from genetic mutations, splice variants, and PTMs is, at any one time, fundamentally limited by *N*. Equation (9) estimated 9 x 10^9^ proteoforms at a given moment in time per HeLa cell [50].

(7) P=N

where *P* and *N* denote proteoforms and protein molecules, respectively.

The number of cysteine-proteoforms (*Ni*) equals the number of cysteine-residue containing proteins in the biological reference frame. Equation (8) estimated 1.70 x 10^9^ cysteine proteoforms per HeLa cell. This value exceeded the unique number (4.04 x 10^7^) by 2-logs because it included duplicates, which were discarded when calculating the unique number.

(8) $$N_{i}=N_{c}$$

where *N*_*i*_ and *N*_c_ are the number of cysteine proteoforms and protein molecules with at least one cysteine residue, respectively. Ergo, *N*_*i*_ is inextricably tied to *N*_*c*_.

Equation (8) invariably constrained any *n*-x dimensional space, limiting the entire cysteine redox phase space. Irrespective of whether *n*-2 or *n*-50 is operated as the *n*-dimensional value in equation (1), the final result is 1.70 x 10^9^ when *N*_*i*_ must equal *N*_*c*_. And, *Ni* remains constant whatever form the sampling of unique proteoforms in the biological accessible *i*-space takes.

In sum, *N* immutably constrains *P* in any given biological reference frame at a given moment in time. Their formal equivalence means *N*_*c*_ sets *N*_*i*_.

### 2.7 Nonlinear rules govern cysteine proteoform dynamics

While *N*_*c*_ caps *N*_*i*_ at any one time, more *N*_*i*_ species can form over time according to the mathematical rules that govern proteoform dynamics. In binary (*n*-2-dimensional) redox phase space, the rules that govern cysteine proteoform dynamics over n-time-steps are.

1. The degrees of freedom (*df*), number of moves that are possible per step, equals the number of redox grades. For example, for *R*-3 + 1 = 4. So, *df* = 4. Hence, a 000 molecule can remain at 000 or move to one of 100,110,101 in one step per rule 2.
2. Only stepwise, plus or minus 1, moves between redox grades can occur. For example, an *R* = 3 molecule cannot go from 100 % to 0%-oxidised in one step.
3. Binary, 100%-reduced or 100%-oxidised, proteoforms can only be acted on in one direction. For example, 000 can only be acted on in an oxidative direction because it cannot be more reduced than it already is.
4. Site-information is conserved. For example, a 011 proteoform can only transition to a 111, 001, or 010 in one step.

These rules are nonlinear because the nature of the moves is determined by the redox grade of the molecule. For example, each unique proteoform is bounded by a unique set of allowed and barred moves (Table 4). Moreover, there are stepwise and asymmetric transitions. Time-and context-dependences are created by barring molecules from jumping across the redox grades in one step. That only oxidising moves are possible for a 000 proteoform creates asymmetry. Site-information establishes a local-dependence on the structure of the proteoform for determining what can happen next. The 010 oxiform cannot move to 001 in one step. The rules establish a dependence on the initial conditions. One would expect a different set of outcomes form varying the initial condition.

**Table 4:** Summary of the input variable used in each Monte Carlo simulation. The bold text denotes one or more values that were manipulated in the run.

Run	*P*_*RED*_ Cys215	*P*_*OX*_ Cys215	*P*_*RED*_ Cys-other	*P*_*OX*_ Cys-other
1	0.9	0.1	0.1286	0.028
2	0.9	**0.0111**	0.1286	**0.00311**
3	0.9	**0.00111**	0.1286	**0.000311**
4	**0.35**	0.1	0.1286	0.028
5	**0.3**	0.1	0.1286	0.028
6	0.3	**0.15**	0.1286	0.028
7	0.3	**0.0510**	0.1286	**0.00311**
8	0.3	**0.00510**	0.1286	**0.000311**
9	0.3	**0.125**	0.1286	**0.028**

In a linear system, such as when describing a single cysteine in binary redox phase space, transitions across the full redox grade are direct and unconstrained. The system moves from fully reduced to oxidised in a single step, representing a 100 % change in redox state. Similarly, the reverse transition is equally direct. Such transitions lack dependencies or constraints imposed by other sites, and the absence of barred transitions ensures that each move is deterministic and proportional.

In a nonlinear dynamical proteoform system, transitions are governed by probabilistic rules, constrained pathways, and site-specific dependencies. Movement between redox grades is stepwise with varying allowed and barred transitions. As a result, the system exhibits emergent properties, requiring advanced mathematical tools like Monte Carlo methods to model the inherently nonlinear, context-dependent *i*-space.

The rules of cysteine proteoform dynamics provide a mathematical framework for understanding redox homeostasis as a dynamical system, elaborating on Sies’ concept of oxidative stress [[52], [53], [54], [55], [56]]. This interpretation introduces new ideas.

1. **Chaotic Properties:**
  o The dynamic system's sensitivity to initial conditions, feedback loops, and asymmetric transitions mirrors other chaotic systems.
2. **Feedback with Other Non**l**inear Systems**:
  o Redox dynamics may interact with other biological phenomena, such as liquid-liquid phase transitions [57,58], which are similarly sensitive to boundary conditions and initial states [59,60].
  o These feedback loops suggest that redox regulation may integrate with broader cellular phenomena, linking processes like phase separation and ageing [[61], [62], [63], [64]].
3. **Basin Attractors**:
  o Viewing cysteine proteoforms as a dynamical system raises the possibility of basin attractors, regions in the *i*-space toward which the system gravitates. These attractors might explain the variability and resilience observed in redox regulation under stress, such as exercise [[65], [66], [67], [68]].

In sum, the rules governing cysteine proteoform dynamics are nonlinear, meaning a given input may not necessarily produce a proportional output.

### 2.8 The oxiform conjecture: A broader set of PTP1B-specific proteoforms are formed than previously imagined

To implement the rules that govern cysteine proteoform dynamics, we computationally explored the “band 2” conjecture [30]. The conjecture derives its moniker from the notion that specific oxidation of a selected site would produce a 2nd mobility-shift band, corresponding to the one oxidised cysteine for the protein of interest, appearing above the 1st 100%-reduced band in a polyethylene glycol (PEG) payload-based immunoblot assay [[69], [70], [71], [72]]. In this regard, the redox regulation (*f*) of human PTP1B (*R* = 10, *n* = 2, *i* = 1024) would define a linear map from point *A* and *B*. Point *A* and *B* represent 0000000000 (0%-oxidised, band 1) and 000100000 (10%-oxidised, band 2), respectively, with Cys215 as position 4. That is, *f* = *B*. To explore this conjecture across the entire PTP1B-specific *i*-space, as enumerated in a matrix, we iterated Monte Carlo simulations [73,74].

The Monte Carlo simulations captured the inherently probabilistic nature of nonlinear redox dynamics by using *P*_*OX*_ and *P*_*RED*_ values. These values capture the probability that a given cysteine will be acted on in the reducing or oxidising direction, distilling a number of complex variables, from electrostatic, kinetic, to structural considerations [75,76], to tunable terms. For example, if a cysteine was always buried, then the probability that it would be oxidised is zero.

While the *P*_*OX*_ and *P*_*RED*_ values were iterated (Table 4), the *P*_*OX*_ value for Cys215 was higher than the other cysteines to reflect its perceived preferential oxidation [30]. Similarly, the Cys215-*P*_*RED*_ value was also higher to reflect it being preferentially acted on by the reductive apparatus of the cell [[77], [78], [79]]. In any case, these *P*-values are not absolute, meaning there is no true value that could be applied. Instead, they evolve dynamically based on the local biochemical environment. For example, the probability that Cys215 is oxidised may temporarily be 0 when its immediate vicinity is devoid of ROS, highlighting the stochastic nature of cysteine proteoform dynamics.

Given the computational demands (2.1 x 10^8^ computations per run), all other cysteines were treated equally with uniform *P*_*RED*_ and *P*_*OX*_ values. After setting the initial condition as point *A* (0%-oxidised, 0000000000), we evolved 70,000, a relevant single cell *N*, individual PTP1B molecules over 300 steps (5 min) according to the rules of proteoform dynamics. For example, 0000000000 cannot be further reduced. The output files recorded the number of molecules in each *i*-state at step 300 (supplementary data file 9). The number of steps was selected because of the aforementioned computational demands and 5-min is a relevant time for redox regulation to occur, such as in the context of growth factors [80].

**Fig. 6:**
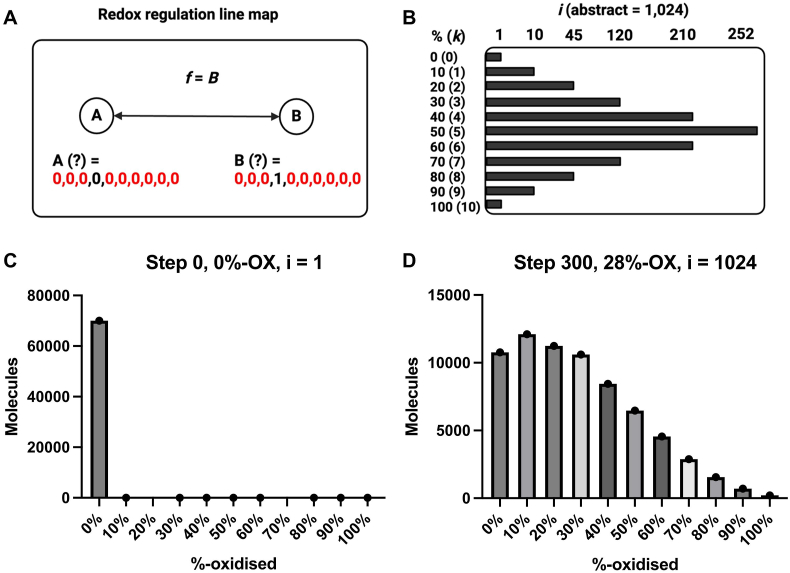
The redox regulation of PTP1B. Top. A. Schematic displaying the linear line map model of PTP1B redox regulation where the function (*f*) is a transition between point A and B, as specified in binary proteoform coordinates with Cys215 at position 4. B. To explore this model, the iterated Monte Carlo simulations considered the full *k*-space distributed *i*-space for human PTP1B. C. In each run, 70,000 PTP1B molecules were initialised in the 0%-oxidised state. As a result, the redox state of the population was 0%-oxidised. And, only one *i*-state was sampled. D. This histogram plot shows the number of molecules in each percentage redox grade at the end of the run #6 (step 300). In this run, the final state of the PTP1B population was 28%-oxidised, the entire *i*-space was sampled. All 1024 forms were present in the final distribution. The 0001000000 proteoform accounted for only 22.2 % of the observed Cys215-oxidation (19 %), meaning a broader set of proteoform coordinates may map to the redox regulation of PTP1B than previously imagined.

Carefully iterating the *P*-values enabled our simulations to capture a variety of perturbations from the initial condition (Fig. 6). Resulting in the weighted mean cysteine redox state of the 10^4^ molecule PTP1B population ranging from 0.08 to 28.81%-oxidised (difference = 28.73 %) in run 3 and 6, respectively. PTP1B-inactivation (*f*), the number of proteoforms where Cys215 was in the 1 state, ranged from 0.01 % to 18.93 % (difference = 18.92 %) in run 3 and 6, respectively. In three runs (#5: 8.25 %; #6: 18.93 %; and #9: 13.36 %), *f* reached a value consistent with the inactivation of PTP1B in a cell signalling context. In support of the utility of the model, these Cys215 percent oxidation values agreed with empirical values, acquired over similar time intervals [10,21,81,82].

**Fig. 7:**
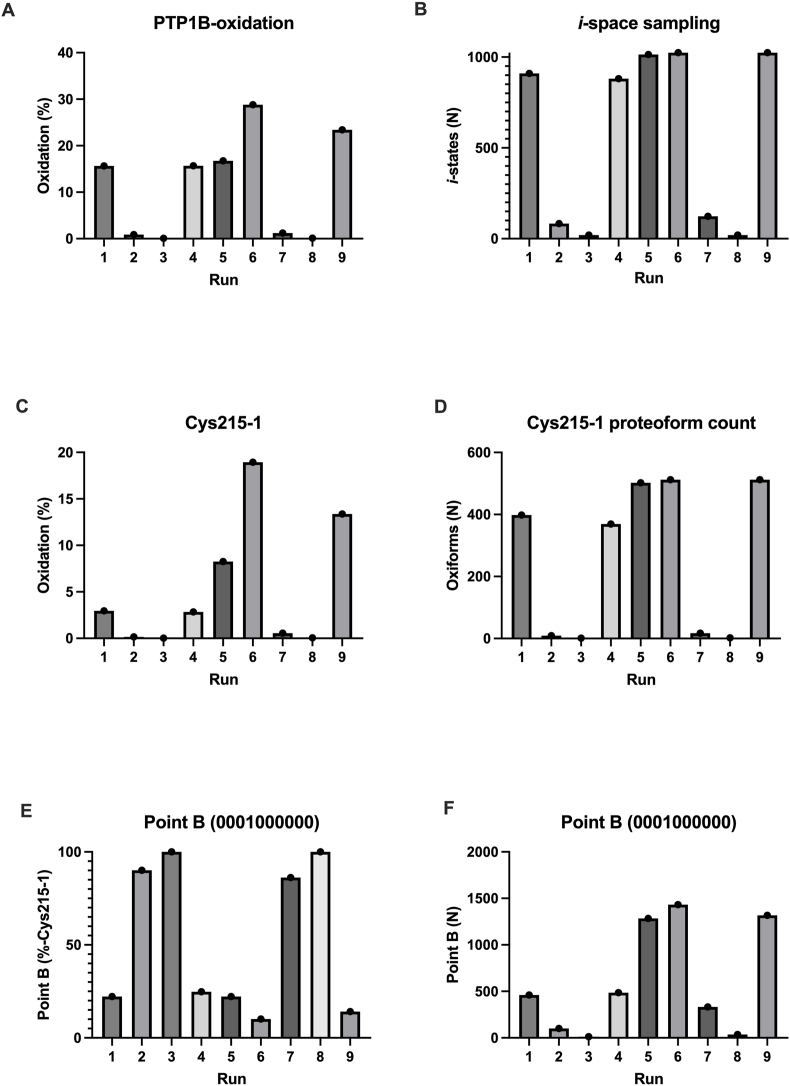
Plots illustrating various PTP1B oxidation indices by run (step 300). A = PTP1B oxidation. B = *i*-space sampling. C = Cys215 oxidation (%). D = Number of proteoforms with Cys215 in the oxidised state (1). E. Percentage of point B as a function of the number of Cys215-1 molecule. F. Number of molecules in point B.

Did 0001000000 fully explain *f*? No (Fig. 7). The 0001000000 proteoform accounted for 22.2 % (1284 molecules), 10.1 % (1432 molecules), and 14.1 % (1317 molecules) of the Cys215-1 molecules in run 5, 6, and 9, respectively. The majority of molecules (always over 75 %) in the Cys215-1 state were in the 20%-oxidised to 100%-oxidised range. Specifically, 502 (49 % of *i*-space), 512 (50 % of *i-*space), and 512 (50 % of *i*-space) proteoforms with Cys215 in the 1 state were formed in run 5, 6, and 9, respectively. In each run, *f* mapped back to a set of proteoform coordinates that were 2-logs larger than previously envisaged.

While the model is a necessarily simplification as, for example, spatial separated pools of PTP1B could have been modelled or the other cysteines could have been treated heterogeneously, our simulations successfully modelled cysteine proteoform dynamics. In support of the utility of the model, 90 % and 100%-oxidised PTP1B oxiforms were observed in an insulin signalling context using the PEG-based immunoblotting assay [83]. Hence, we propose the “oxiform conjecture”, which posits that many redox regulatory events are associated with the formation of highly oxidised *i*-states. Their formation would result in a nontrivial sampling of thetheoretical *i*-space.

In sum, Monte Carlo simulations supported the idea that the redox regulation of PTP1B may map back to a broader set of proteoform coordinates, 512 vs 1 *i*-states, than previously imagined. These results support the broader conjecture that oxiforms bearing multiply oxidised cysteines are biologically important. We suggest that they explain some features of emergent complexity, such as cysteine oxidation hierarchies and dependencies.

### 2.9 Visualising proteoform-specified cysteine oxidation hierarchies and dependencies

**Fig. 8:**
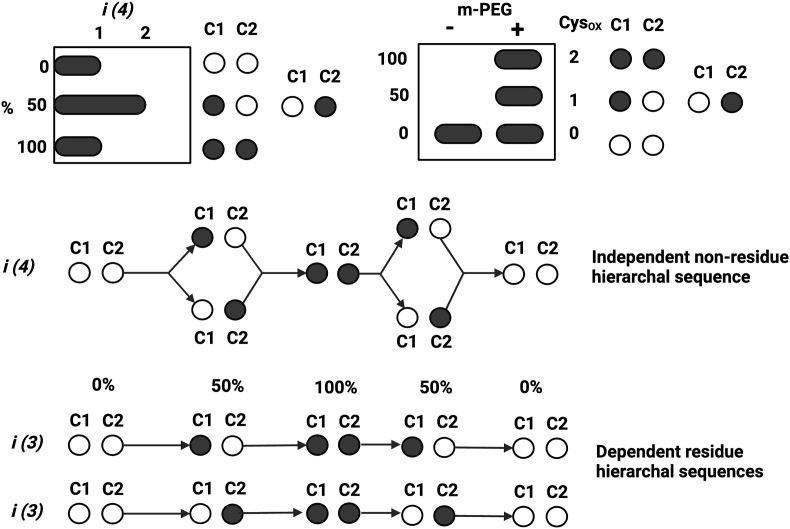
The top left plot depicts the distribution of unique cysteine redox proteoforms (*i*) for a protein with 2 cysteine residues (*n*^*R*^ = 4), such as ATP5A, across the three mathematically allowed percentage redox grades. The white and black circles represent reduced and oxidised cysteines residues (C1: Cys244 or C2: Cys294), respectively. The top right plot depicts an outcome of a maleimide-conjugated polyethylene glycol (PEG)-payload (m-PEG) immunoblot assay. Without m-PEG, all 4 proteoforms migrate at an identical apparent molecular mass. With m-PEG, each oxiform, a proteoform containing at least one oxidised cysteine, is electrically mobility-shifted to an extent that depends on the number of oxidised cysteine residues. The 100%-reduced and 100%-oxidised binary bands are cysteine residue specified. The lower panel displays cysteine redox proteoform diagrams (CPD). They illustrate the cysteine residue independent and dependent pathways in binary redox phase space. In the independent pathway (top CPD), the oxidation of C1 is not conditional upon the oxidation of C2 and vice versa. With no hierarchy between the residues, all the biologically accessible paths between the different percentage redox grades can be explored. In dependent pathways, the stepwise movement between percentage redox grades is conditional upon the oxidation of C1 (middle CPD) or C2 (lower CPD). By introducing hierarchal dependence into the sequence conditional rules generate preferential oxidation pathways that minimise the accessed *i*-space.

To visualise proteoform specific cysteine oxidation hierarchies and dependencies, we created an app for generating cysteine proteoform diagrams (CPD_generator). These diagrams abstract theoretical pathways of redox transitions across percentage redox grades. They provide a resource for explaining experimental observations, as well as, generating and testing hypotheses relevant to proteoform-centric theories of redox regulation. To spotlight a CPD, we plotted the *i*-space in a histogram and immunoblot format for human ATP5A, a redox regulated protein [84]. Since every redox graded band is sampled in *X. laevis* oocytes [[85], [86], [87]], we defined the paths to the ATP5A-specific proteoform bands for the two conserved cysteines Cys244 (C1) and Cys294 (C2) in the CPD (Fig. 8).

In the independent pathway (top CPD), C1 oxidation is not reliant C2 oxidation and vice versa. Independence means all of the possible paths between the distinct %-*i*-space can be explored, maximising the biologically accessed *i*-space. The implications for *f* in redox binary are:

*f* = C1_1_/C2_0_ OR C1_0_/C2_1_ where *f* depends on just the 50 % redox grade (i.e., *f* is cysteine residue agnostic).

*f* = C1_1_ & C2_1_ via either 50 % redox grade pathway where *f* depends on the 100 % redox grade (i.e., there is no preferred route to *f*).

In the dependent pathway, stepwise movements in *k*-space depend upon the oxidation of C1 (middle CPD) or C2 (lower CPD). Hierarchal cysteine oxidation dependencies generate preferred pathways that minimise the biologically accessed *i*-space. The implications for *f* in redox binary are:

*f* = C1_1_/C2_0_ where *f* depends on just the 50 % redox grade (i.e., *f* is cysteine residue dependent).

*f* = C1_1_ & C2_1_ via C1_1_/C2_0_ 50%-redox grade pathway where *f* depends on the 100 % redox grade (i.e., *f* is route dependent).

Stepwise reductive movements in the *i-*space can be dependent or independent. Different rules may operate in the “oxidative” and “reductive” branches of the CPD path space. For example, the forward “oxidative direction” path to 100%-oxidised proteoform may rely on C1 priming C2 oxidation but the backward “reductive direction” path to the 100%-reduced proteoform may be independent. The initial condition may be 50%-oxidised, with cyclical up or down steps from there. There could be distinct initial condition setpoints for different pools of the same molecule. Like the strings of an instrument, setpoints may constrain the “notes” that otherwise identical molecules play, affording more or less access to a proteoform-based redox “key” range—*i*-states. Akin to a redox capo on a cysteine guitar.

## 3 Discussion

Our work introduces a proteoform-centric theory of redox regulation, fundamentally advancing the traditional site-centric paradigm. This theory conceptualises the *i*-space as a theoretical landscape of all possible cysteine proteoforms and defines the dynamics of these proteoforms. Integrating probability and nonlinear rules distilled the complex biological and biochemical inputs controlling cysteine oxidation, from electrostatics to kinetics [75,88,89], into adjustable *P*-values. This advance suggested a tuneable probabilistic model of redox regulation. This framework integrates theoretical predictions, computational modelling, and empirical validation, establishing a robust platform for exploring redox regulation at the proteoform level.

This work presents a framework where stepwise transitions, site dependencies, and asymmetries in redox transitions underpin the nonlinear rules of redox regulation. These nonlinear rules of a dynamical system introduce the potential for chaotic behaviour, which has already been demonstrated in a cysteine-dependent bacterial response to oxidative stress [90]. Considering chaos can connect redox regulation to information theory [91,92] (which is ultimately what is being relayed in cell signalling), offering explanations for certain system states being preferred and then perturbed through the lens of basin attractors [93]. Information theory would be particularly productive route to follow with regards to understanding dysregulated redox regulation in the brain [[94], [95], [96]]—the ultimate biological information processor [97]. Despite all their biochemical differences with respect to cysteine oxidation (e.g., pH, kinetics, etc.), the redox profiles of proteins in the same *R* class may display self-similar fractal oxidation patterns. Or fractal-like branches are created as a population of molecules sample their *i*-space over time, which might be captured with recursive equations that have yet to be developed. Hence, chaos theory provides an attractive theoretical lens for exploring redox regulation, especially the interplay with other dynamical systems like condensates [98].

The theoretical *i*-space introduces the idea of an abstract mathematical plane housing all of the possible *i*-states that a given protein or set of proteins can adopt. Abstraction allows us to imagine each molecule in a hypothetical superimposition of all *i*-states, such as [00,10,01,11] for the *R* = 2 class in binary redox phase space. On a biological plane, rules govern what *i*-states can exist. First, only expressed molecules can occupy an *i*-state. Second, the full theoretical *i*-space can only be accessed when there are enough protein molecules (*N*) in the system to accommodate each *i*-state. Third, out of the biologically accessible *i*-space, each molecule can only be in one *i*-state at a time. Every protein molecule with a cysteine is in an *i*-state. Finally, rule-based probabilities govern the *i*-state that a molecule is in at a particular moment in time and over time. The probability of each *i*-state is influenced by a complex set of considerations, from biochemical to structural. Moreover, these probabilities dynamically evolve over time. For example, the *P*-space would evolve when a molecule travels from a “reducing” to an “oxidising” nanodomain [99,100]. Relatedly, the “history” of the molecule shapes what happens next, precluding some reactions while favouring others [28].

The “oxiform conjecture” suggests that redox regulation is associated with the formation of highly-oxidised oxiforms. In support, Monte Carlo simulations of PTP1B demonstrated the emergence of these higher-order states and their role in expanding the proteoform coordinates associated with redox regulation. Notably, these simulations revealed that traditional site-centric models may appreciably underestimate the complexity and diversity of cysteine proteoform dynamics, which may involve the nontrivial sampling of the *i*-space. The sampling of this *i*-space may in some cases be associated with distinct cysteine oxidation dependencies and hierarchies. Even when the major redox regulatory function is executed by a site, it is possible, perhaps even likely in many cases, that the other cysteines shape the nature of the redox regulation event. This may conceptually occur in many ways. For example, by competing with one or more sites for oxidation, other residues can suppress the signal. Reciprocally, when they are randomly acted on by reductants, they can amplify the signal. The proteoform-centric viewpoint can describe and model these nuanced emergent complexities.

**Fig. 9:**
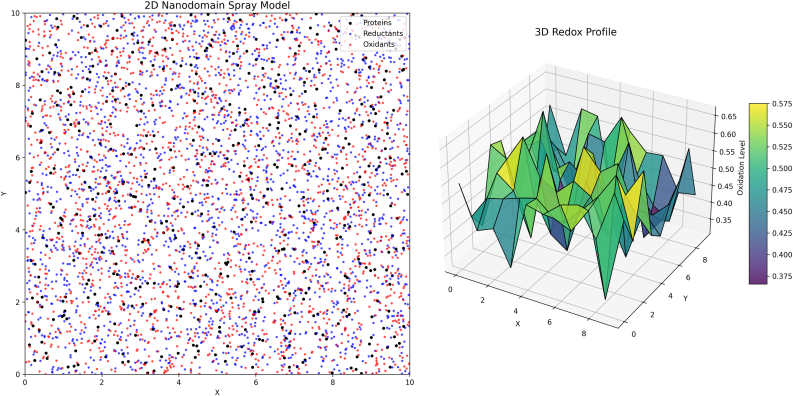
The spray model of redox regulation. The 2D model on the left depicts proteins (black), reductants (blue), and oxidants (red) heterogeneously distributed in space. The net result of these localised asymmetries yields a protein molecule oxidation profile with peaks and troughs as a function of space, as illustrated by the 3D redox profile plot generated from the 2D nanodomains. Some of these nanodomains are proposed to support the formation of highly oxidised oxiforms. In certain instances, they will from as function of the molecule being indiscriminately acted on by oxidants in a “spray” like fashion. The result is a broader sampling of the *i*-space than might otherwise have been envisaged. The figure was automatically generated using the Spray_model.py script.

This suggests a “spray” model of redox regulation. In one iteration of the spray model, a set of protein molecules are distributed heterogeneously in nanodomains—discrete small regions of space. Superimposed on this, is the heterogeneous spatial partitioning of cellular reductants and oxidants across these spaces. The net result is that the molecule will be more oxidised in some nanodomains compared to others, which is consistent with the work of Kritsiligkou and co-workers [99]. The spray moniker is derived from the idea that in the oxidising nanodomains the molecule is indiscriminately acted on by oxidising agents—quite distinct from a selective laser-like action. This sets the model apart from others that seek to account for site-selective cysteine oxidation via kinetically more reactive oxidants, transfer of oxidising equivalents via relays, or temporary inactivation of key enzymes [[101], [102], [103]]. The significance of the spray model for our conjecture is the formation of oxiforms, some of which would then modulate said oxidation. The outcome is a 3D oxiform profile with peaks and troughs (Fig. 9).

Looking forward, it will be important to understand the interplay between cysteine proteoforms and other PTMs like phosphorylation. The quantitative treatment of the theoretical *i*-space, as outlined in Section 2.1, can be generalised to other PTMs, enabling the calculation of biologically accessible PTM-space for residues, such as methionine [104]. This opens up opportunities to study how redox proteoforms interact with other PTMs to influence biological processes, potentially leading to heterogeneity due to their variability [26,27,105]. For example, as proposed in the spray model, highly oxidising nanodomains could facilitate the coincident occurrence of cysteine oxidation and indices of oxidative damage like tyrosine nitration [106], potentially in the context of cell signalling. These intersections between redox proteoforms and other PTMs, as well as potential interactions with splice variants and SNPs, highlight the need for further exploration into how such modifications collectively shape biological responses.

When interpreting our results, some limitations must be borne in mind. First, as a proteoform-centric discourse, we omitted low-molecule weight thiols like glutathione. Second, we did not consider variability about the human proteome or protein complexes [107,108]. Note, we expect our human theoretical *i*-space value to be robust to updates of the reference proteome due to the scale-free nature of the space. A protein with over 500 cysteines would need to be discovered to make any appreciable difference to our calculations. A protein complex ensemble *i*-space will increase as an aggregate of its unitary terms. For example, each GAPDH tetramer would have an *i*-space of 4096 *i*-states in aggregate compared to only 8 *i*-states for each monomeric unit. Third, some of our reasoning only considered binary redox phase space. Despite the explanatory power of the binary model, it cannot factor in the influence of specific chemotypes. For example, the formation of a thiyl radical may shape local *P*-values for other cysteines by reacting with glutathione, then oxygen to form superoxide [[109], [110], [111]]. Elucidating the chemotypes that describe the oxidised state is essential for unravelling the function of a particular *i*-state or set thereof, which cannot be deduced from the binary structure of an *i*-state alone.

To conclude, this study formalised a theoretical description of redox regulation at the proteoform level. At the proteoform level, redox regulation can emerge from the interplay of probabilistic transitions, hierarchical dependencies, and proteoform diversity. This proteoform-centric framework is poised to guide future investigations into redox biology.

## 4 Methods

### 4.1 Coding

Unless otherwise stated, computational analyses were coded in Python and implemented in a Google Colaboratory (Colab) environment, which hosts virtual Jupyter notebooks. Our source code is available via the Cystine-i-cloud GitHub repository [112], inclusive of requirements.txt, readme, and MIT licence files. The MIT licence enables the code to be freely modified and used, provided it is cited using the Zendo DOI.

### 4.2 Calculating the theoretical *i*-space

To calculate the theoretical *i*-space, reference proteomes (Table 5) were downloaded from Uniprot as gzipped FATSA files, which were uploaded into a Colab workspace where the scriptcys1.py code was implemented. The script pre-processed the files by reading the gzipped FASTA file and extracting the amino acid sequences. Header lines, which started with the '>' character, were skipped to ensure only the sequence data were processed. Each line was stripped of trailing newlines or spaces. After importing the necessary libraries, the script.

1. Parsed the gzip file and extract protein sequences.
2. Counted the number of cysteine residues ('C') in each protein sequence.
3. Listed the number of proteins with different counts of cysteine residues.
4. Exported the results to an Excel file with two columns: column A for cysteine residues and column B for the number of proteins.

The Excel file was processed by implementing a “PERMUTATONIA” function to solve equation (1) as “*i space*” for each “*cysteine residue*” value. For example, 2 = 4. Equation (2) was implemented by multiplying “*i space*” by “*proteins*” (e.g., 4 x 10 = 40). The resultant values were summed to calculate the theoretical *i*-space.

### 4.3 Calculating the theoretical PTM-space

The theoretical PTM-space of proteins was calculated by parsing a gzipped FASTA file containing UniProt entries. For each protein, the number of amino acids was counted, which replaced *R* in equations (1), (2)), and the PTM-space was computed as in *n* = 2-dimensional space. Results, including UniProt entries, amino acid counts, and PTM-space (in scientific notation), were saved to an Excel file. The cumulative PTM-space across all proteins was calculated in log-space to handle large values efficiently. In another iteration of the script, the *n*-dimensional space was adjusted to account for PTM depth. To do so, we implemented the PTM_space_cal.py and PTM_depth_cal.py scripts, respectively.

### 4.4 Defining the composition of the human *i*-space

To define the composition of the human *i*-space, the scriptcys1.py script output file (supplementary data file 1) was used to create the visualisations displayed in Fig. 2. To retrieve the identity of the protein with the most cysteine residues, we used the Cys_R_integer_find.py script. Supplementary data file 1 were manually analysed to create Table 2 by sorting the data using cysteine classifier ranges.

### 4.5 Mathematically limiting the biological accessible *i*-space

To mathematically limit the biologically accessible *i*-space, equation (3) constrained the number of “downloadable” cysteine proteoforms using a protein expression argument. To apply equation (3), the Cys_Expression.py script was implemented on empirical data (Supplementary Table S2 from Ref. [45]) by calculating the *i*-space for each protein before summing them.

After designing it to limit the biologically accessible *i*-space using a copy number (*N*) argument, we implemented equation (4) on empirical data (Supplementary Table S7 from Ref. [45]). The Cys_Expression.py was implemented to assign a cysteine residue integer value to each *N* value before an *i*-space was derived. An Excel “MIN” function was used to select the minimum value of *N* or i-space, which were summed to compute the biologically accessible *i*-space.

### 4.6 Sampling the *i*-space

For the results described in section 2.6, the *i*-space of an empirically-derived dataset was iteratively sampled under different conditions and assumptions using the Sampling_i_space.py, sampling_i_space_random.py. For example, latter script models the distribution of protein molecules across cysteine oxidation space for cysteine-containing proteins. Proteins are ranked by abundance with the top 3000 most abundant molecules set in the super-Poisson reduced state. The remaining molecules were assigned to percent oxidised redox grades based on predefined profiles (e.g., random, Gaussian, or super-Poisson). Key metrics, such as minimum and maximum accessible proteoforms, redox state, and total proteome redox state, are calculated. Results, including distribution profiles and weighted redox states, are saved to an Excel file for further analysis.

### 4.7 Monte Carlo simulations

Monte Carlo simulations were performed on a Google Cloud N2-highmem-32 virtual machine instance configured with 32-vCPUs, 128-GB RAM, 100-GB balanced persistent disk space, and an Ubuntu 20.04 LTS operating system. After installing the requirements (python3, pandas, numpy, openpyxl, xlsxwriter, google-cloud-storage) in the virtual terminal, the Cysteine-i-cloud repository was cloned and entered using the “cd” command. The Redox_Monte_Carlo.py script was executed using the “Python3 Redox_Monte_Carlo.py” command. In each run, 70,000 PTP1B molecules were initialised in the 0000000000 *i*-state in step 1 and individually evolved over 300 steps according to the probability of oxidation and reduction values provided in Table 5. In each step, the source code used a proteoform matrix to record the *i*-state that each molecule occupied, enabling the final distribution to be recorded in an Excel file. The Excel files were analysed using an automated script.

**Table 5:** Downloaded reference proteomes by species with strain information where applicable.

Species	Reference proteome	Strain
*E. coli*	https://www.uniprot.org/proteomes?query=%28organism_id%3A83333%29+AND+%28proteome_type%3A1%29	Strain K12 (83333)
*S. cerevisiae*	https://www.uniprot.org/uniprotkb?query=yeast&facets=reviewed%3Atrue%2Cmodel_organism%3A559292	strain ATCC 204508/S288c
*C. elegans*	https://www.uniprot.org/proteomes/UP000001940	Bristol N2
*Drosophila*	https://www.uniprot.org/proteomes/UP000000803	Berkeley
*D. rerio*	https://www.uniprot.org/proteomes/UP000000437	n/a
*X. tropicalis*	https://www.uniprot.org/proteomes/UP000008143	n/a
*M. musculus*	https://www.uniprot.org/proteomes/UP000000589	C57BL/6J
*H. sapiens*	https://www.uniprot.org/uniprotkb?query=human&facets=model_organism%3A9606%2Creviewed%3Atrue	n/a

### 4.8 Visualising proteoform-specified codes

To visualise proteoform-specified codes, we created the custom Cys_CPD_code.py script, located in a distinct repository, to run on the streamlit application. To use the application, one inputs an UniProt accession for a protein of interest. The script prints the number of cysteine residues, their positions, and the number of cysteine proteoforms. In addition, the script prints the number of cysteine proteoforms in each percentage redox grade. Finally, the script generates a heatmap visualisation, a CPD, of the cysteine proteoforms. Reduced and oxidised cysteines are displayed in white and black, respectively.

### 4.9 Visualisations

The plots displayed to visualise the data were created in Colab using the Matlab package and exported at 300 DPI. The scripts to make several of the plots from our data or other datasets are available in the cysteine-i-cloud repository. The visualisations displayed in the bottom panel of Fig. 6 and all of Fig. 7 were created using Graphpad Prism v.10.

### 4.10 Summary statistics and statistical analysis

Certain summary values were calculated using automated scripts. For example, the number of cysteines in the human proteome was calculated using the script summaryscript.py. After importing numpy into Colab, most summary statistics, such as mean and median values, were calculated using scripts, such as Cysteinestats.py.

## CRediT authorship contribution statement

**James N. Cobley:** Writing – review & editing, Writing – original draft, Investigation, Formal analysis, Data curation, Conceptualization. **Panagiotis N. Chatzinikolaou:** Writing – review & editing, Visualization, Software. **Cameron A. Schmidt:** Writing – review & editing, Software, Resources, Formal analysis.

## Declaration of competing interest

All authors declare that they have no conflicts of interest.

## Data Availability

The data is attached in the supplementary material and all of the source code is on github.

## References

[1] Moosmann B. (2021). Redox biochemistry of the genetic code. Trends Biochem. Sci..

[2] Sies H., Belousov V.V., Chandel N.S., Davies M.J., Jones D.P., Mann G.E., Murphy M.P., Yamamoto M., Winterbourn C. (2022). Defining roles of specific reactive oxygen species (ROS) in cell biology and physiology. Nat Rev Mol Cell Bio.

[3] Sies H., Mailloux R.J., Jakob U. (2024). Fundamentals of redox regulation in biology. Nat. Rev. Mol. Cell Biol..

[4] Sies H., Jones D.P. (2020). Reactive oxygen species (ROS) as pleiotropic physiological signalling agents. Nat Rev Mol Cell Bio.

[5] Holmström K.M., Finkel T. (2014). Cellular mechanisms and physiological consequences of redox-dependent signalling. Nat Rev Mol Cell Bio.

[6] D'Autréaux B., Toledano M.B. (2007). ROS as signalling molecules: mechanisms that generate specificity in ROS homeostasis. Nat Rev Mol Cell Bio.

[7] Parvez S., Long M.J.C., Poganik J.R., Aye Y. (2018). Redox signaling by reactive electrophiles and oxidants. Chem Rev.

[8] Alcock L.J., Perkins M.V., Chalker J.M. (2017). Chemical methods for mapping cysteine oxidation. Chem. Soc. Rev..

[9] Marinho H.S., Real C., Cyrne L., Soares H., Antunes F. (2014). Hydrogen peroxide sensing, signaling and regulation of transcription factors. Redox Biol..

[10] Antunes F., Brito P.M. (2017). Quantitative biology of hydrogen peroxide signaling. Redox Biol..

[11] Brigelius-Flohé R., Flohé L. (2011). Basic principles and emerging concepts in the redox control of transcription factors. Antioxid Redox Sign.

[12] Rhee S.G. (2006). H2O2, a necessary evil for cell signaling. Science.

[13] Janssen-Heininger Y.M.W., Mossman B.T., Heintz N.H., Forman H.J., Kalyanaraman B., Finkel T., Stamler J.S., Rhee S.G., van der Vliet A. (2008). Redox-based regulation of signal transduction: principles, pitfalls, and promises. Free Radical Bio Med.

[14] Sundaresan M., Yu Z.-X., Ferrans V.J., Irani K., Finkel T. (1995). Requirement for generation of H2O2 for platelet-derived growth factor signal transduction. Science.

[15] Irani K., Xia Y., Zweier J.L., Sollott S.J., Der C.J., Fearon E.R., Sundaresan M., Finkel T., Goldschmidt-Clermont P.J. (1997). Mitogenic signaling mediated by oxidants in ras-transformed fibroblasts. Science.

[16] Zheng M., Åslund F., Storz G. (1998). Activation of the OxyR transcription factor by reversible disulfide bond formation. Science.

[17] Storz G., Tartaglia L.A., Ames B.N. (1990). Transcriptional regulator of oxidative stress-inducible genes: direct activation by oxidation. Science.

[18] Choi H.-J., Kim S.-J., Mukhopadhyay P., Cho S., Woo J.-R., Storz G., Ryu S.-E. (2001). Structural basis of the redox switch in the OxyR transcription factor. Cell.

[19] van Montfort R.L.M., Congreve M., Tisi D., Carr R., Jhoti H. (2003). Oxidation state of the active-site cysteine in protein tyrosine phosphatase 1B. Nature.

[20] Salmeen A., Andersen J.N., Myers M.P., Meng T.-C., Hinks J.A., Tonks N.K., Barford D. (2003). Redox regulation of protein tyrosine phosphatase 1B involves a sulphenyl-amide intermediate. Nature.

[21] Meng T.-C., Buckley D.A., Galic S., Tiganis T., Tonks N.K. (2004). Regulation of insulin signaling through reversible oxidation of the protein-tyrosine phosphatases TC45 and PTP1B. J. Biol. Chem..

[22] Londhe A.D., Bergeron A., Curley S.M., Zhang F., Rivera K.D., Kannan A., Coulis G., Rizvi S.H.M., Kim S.J., Pappin D.J., Tonks N.K., Linhardt R.J., Boivin B. (2020). Regulation of PTP1B activation through disruption of redox-complex formation. Nat. Chem. Biol..

[23] Paulsen C.E., Carroll K.S. (2013). Cysteine-Mediated redox signaling: chemistry, biology, and tools for discovery. Chem Rev.

[24] Shi Y., Carroll K.S. (2020). Activity-based sensing for site-specific proteomic analysis of cysteine oxidation. Accounts Chem. Res..

[25] Smith L.M., Kelleher N.L., Linial M., Goodlett D., Langridge-Smith P., Goo Y.A., Safford G., Bonilla L., Kruppa G., Zubarev R., Rontree J., Chamot-Rooke J., Garavelli J., Heck A., Loo J., Penque D., Hornshaw M., Hendrickson C., Pasa-Tolic L., Borchers C., Chan D., Young N., Agar J., Masselon C., Gross M., McLafferty F., Tsybin Y., Ge Y., Sanders I., Langridge J., Whitelegge J., Marshall A. (2013). Proteoform: a single term describing protein complexity. Nat. Methods.

[26] Smith L.M., Kelleher N.L. (2018). Proteoforms as the next proteomics currency. Science.

[27] Aebersold R., Agar J.N., Amster I.J., Baker M.S., Bertozzi C.R., Boja E.S., Costello C.E., Cravatt B.F., Fenselau C., Garcia B.A., Ge Y., Gunawardena J., Hendrickson R.C., Hergenrother P.J., Huber C.G., Ivanov A.R., Jensen O.N., Jewett M.C., Kelleher N.L., Kiessling L.L., Krogan N.J., Larsen M.R., Loo J.A., Loo R.R.O., Lundberg E., MacCoss M.J., Mallick P., Mootha V.K., Mrksich M., Muir T.W., Patrie S.M., Pesavento J.J., Pitteri S.J., Rodriguez H., Saghatelian A., Sandoval W., Schlüter H., Sechi S., Slavoff S.A., Smith L.M., Snyder M.P., Thomas P.M., Uhlén M., Eyk J.E.V., Vidal M., Walt D.R., White F.M., Williams E.R., Wohlschlager T., Wysocki V.H., Yates N.A., Young N.L., Zhang B. (2018). How many human proteoforms are there?. Nat. Chem. Biol..

[28] Cobley J.N. (2023). Oxiforms: unique cysteine residue‐ and chemotype‐specified chemical combinations can produce functionally‐distinct proteoforms. Bioessays.

[29] Cobley J.N. (2023). 50 shades of oxidative stress: a state-specific cysteine redox pattern hypothesis. Redox Biol..

[30] Cobley J.N. (2024). Exploring the unmapped cysteine redox proteoform landscape. Am. J. Physiol.-Cell Physiol..

[31] Jones D.P. (2024). Redox organization of living systems. Free Radic. Biol. Med..

[32] Jones D.P., Sies H. (2015). The redox code. Antioxid Redox Sign.

[33] Cobley J.N., Margaritelis N.V., Chatzinikolaou P.N., Nikolaidis M.G., Davison G.W. (2024). Ten “cheat codes” for measuring oxidative stress in humans. Antioxidants.

[34] J.N. Cobley, A. Noble, M. Guille, Cleland Immunoblotting Unmasks Unexpected Cysteine Redox Proteoforms, (n.d.). 10.1101/2024.09.18.613741.

[35] Halliwell B., Gutteridge J. (2015).

[36] Miseta A., Csutora P. (2000). Relationship between the occurrence of cysteine in proteins and the complexity of organisms. Mol. Biol. Evol..

[37] Mu B., Zeng Y., Luo L., Wang K. (2024). Oxidative stress-mediated protein sulfenylation in human diseases: past, present, and future. Redox Biol..

[38] Kitamura N., Galligan J.J. (2023). A global view of the human post-translational modification landscape. Biochem. J..

[39] Wensien M., von Pappenheim F.R., Funk L.-M., Kloskowski P., Curth U., Diederichsen U., Uranga J., Ye J., Fang P., Pan K.-T., Urlaub H., Mata R.A., Sautner V., Tittmann K. (2021). A lysine–cysteine redox switch with an NOS bridge regulates enzyme function. Nature.

[40] Barabási A.-L. (2009). Scale-free networks: a decade and beyond. Science.

[41] Dietz K.-J., Hell R. (2015). Thiol switches in redox regulation of chloroplasts: balancing redox state, metabolism and oxidative stress. Biol. Chem..

[42] Pillay C.S., Rohwer J.M. (2024). Computational models as catalysts for investigating redoxin systems. Essays Biochem..

[43] Sies H. (2024). Dynamics of intracellular and intercellular redox communication. Free Radic. Biol. Med..

[44] Ulrich K., Jakob U. (2019). The role of thiols in antioxidant systems. Free Radical Bio Med.

[45] Nagaraj N., Wisniewski J.R., Geiger T., Cox J., Kircher M., Kelso J., Pääbo S., Mann M. (2011). Deep proteome and transcriptome mapping of a human cancer cell line. Mol. Syst. Biol..

[46] Hansen R.E., Roth D., Winther J.R. (2009). Quantifying the global cellular thiol–disulfide status. Proc National Acad Sci.

[47] Gatto L., Aebersold R., Cox J., Demichev V., Derks J., Emmott E., Franks A.M., Ivanov A.R., Kelly R.T., Khoury L., Leduc A., MacCoss M.J., Nemes P., Perlman D.H., Petelski A.A., Rose C.M., Schoof E.M., Eyk J.V., Vanderaa C., Yates J.R., Slavov N. (2023). Initial recommendations for performing, benchmarking and reporting single-cell proteomics experiments. Nat. Methods.

[48] Xiao H., Jedrychowski M.P., Schweppe D.K., Huttlin E.L., Yu Q., Heppner D.E., Li J., Long J., Mills E.L., Szpyt J., He Z., Du G., Garrity R., Reddy A., Vaites L.P., Paulo J.A., Zhang T., Gray N.S., Gygi S.P., Chouchani E.T. (2020). A quantitative tissue-specific landscape of protein redox regulation during aging. Cell.

[49] Boatner L.M., Palafox M.F., Schweppe D.K., Backus K.M. (2023). CysDB: a human cysteine database based on experimental quantitative chemoproteomics. Cell Chem. Biol..

[50] Milo R. (2013). What is the total number of protein molecules per cell volume? A call to rethink some published values. Bioessays.

[51] Carbonara K., Andonovski M., Coorssen J.R. (2021). Proteomes are of proteoforms: embracing the complexity. Proteomes.

[52] Sies H. (2015). Oxidative stress: a concept in redox biology and medicine. Redox Biol..

[53] Sies H. (2021). Oxidative eustress: on constant alert for redox homeostasis. Redox Biol..

[54] Sies H., Berndt C., Jones D.P. (2016). Oxidative stress. Annu. Rev. Biochem..

[55] Sies H. (1985). Oxidative stress.

[56] Sies H. (2019).

[57] Alberti S., Hyman A.A. (2021). Biomolecular condensates at the nexus of cellular stress, protein aggregation disease and ageing. Nat Rev Mol Cell Bio.

[58] Galvanetto N., Ivanović M.T., Chowdhury A., Sottini A., Nüesch M.F., Nettels D., Best R.B., Schuler B. (2023). Extreme dynamics in a biomolecular condensate. Nature.

[59] Dai Y., Chamberlayne C.F., Messina M.S., Chang C.J., Zare R.N., You L., Chilkoti A. (2023). Interface of biomolecular condensates modulates redox reactions. Chem.

[60] Fuentes-Lemus E., Davies M.J. (2023). Effect of crowding, compartmentalization and nanodomains on protein modification and redox signaling – current state and future challenges. Free Radical Bio Med.

[61] Cobley J.N., Moult P.R., Burniston J.G., Morton J.P., Close G.L. (2015). Exercise improves mitochondrial and redox-regulated stress responses in the elderly: better late than never. Biogerontology.

[62] Lane N. (2011). Mitonuclear match: optimizing fitness and fertility over generations drives ageing within generations. Bioessays.

[63] Balaban R.S., Nemoto S., Finkel T. (2005). Mitochondria, oxidants, and aging. Cell.

[64] Moosmann B., Behl C. (2008). Mitochondrially encoded cysteine predicts animal lifespan. Aging Cell.

[65] Margaritelis N.V., Cobley J.N., Nastos G.G., Papanikolaou K., Bailey S.J., Kritsiligkou P., Nikolaidis M.G. (2024). “Unlocking athletic potential: exploring exercise physiology from mechanisms to performance”: evidence-based sports supplements: a redox analysis. Free Radic. Biol. Med..

[66] Margaritelis N.V., Cobley J.N., Paschalis V., Veskoukis A.S., Theodorou A.A., Kyparos A., Nikolaidis M.G. (2016). Principles for integrating reactive species into in vivo biological processes: examples from exercise physiology. Cell. Signal..

[67] Cobley J.N. (2020). Oxidative stress.

[68] Cobley JamesN., Davison G.W. (2022).

[69] Makmura L., Hamann M., Areopagita A., Furuta S., Muoz A., Momand J. (2001). Development of a sensitive assay to detect reversibly oxidized protein cysteine sulfhydryl groups. Antioxid Redox Sign.

[70] van Leeuwen L.A.G., Hinchy E.C., Murphy M.P., Robb E.L., Cochemé H.M. (2017). Click-PEGylation – a mobility shift approach to assess the redox state of cysteines in candidate proteins. Free Radical Bio Med.

[71] Cobley J.N., Sakellariou G.K., Husi H., McDonagh B. (2019). Proteomic strategies to unravel age-related redox signalling defects in skeletal muscle. Free Radical Bio Med.

[72] Cobley J.N., Husi H. (2020). Immunological techniques to assess protein thiol redox state: opportunities, challenges and solutions. Antioxidants.

[73] Metropolis N., Ulam S. (1949). The Monte Carlo method. J. Am. Stat. Assoc..

[74] Kalos M.H., Whitlock P.A. (2022). Monte Carlo methods.

[75] Winterbourn C.C., Hampton M.B. (2008). Thiol chemistry and specificity in redox signaling. Free Radical Bio Med.

[76] Poole L.B. (2015). The basics of thiols and cysteines in redox biology and chemistry. Free Radic. Biol. Med..

[77] Brito P.M., Antunes F. (2014). Estimation of kinetic parameters related to biochemical interactions between hydrogen peroxide and signal transduction proteins. Front. Chem..

[78] Schwertassek U., Haque A., Krishnan N., Greiner R., Weingarten L., Dick T.P., Tonks N.K. (2014). Reactivation of oxidized PTP1B and PTEN by thioredoxin 1. FEBS J..

[79] Dagnell M., Frijhoff J., Pader I., Augsten M., Boivin B., Xu J., Mandal P.K., Tonks N.K., Hellberg C., Conrad M., Arnér E.S.J., Östman A. (2013). Selective activation of oxidized PTP1B by the thioredoxin system modulates PDGF-β receptor tyrosine kinase signaling. Proc. Natl. Acad. Sci..

[80] Behring J.B., van der Post S., Mooradian A.D., Egan M.J., Zimmerman M.I., Clements J.L., Bowman G.R., Held J.M. (2020). Spatial and temporal alterations in protein structure by EGF regulate cryptic cysteine oxidation. Sci. Signal..

[81] Mahadev K., Zilbering A., Zhu L., Goldstein B.J. (2001). Insulin-stimulated hydrogen peroxide reversibly inhibits protein-tyrosine phosphatase 1B in vivo and enhances the early insulin action cascade. J. Biol. Chem..

[82] Lee S.-R., Kwon K.-S., Kim S.-R., Rhee S.G. (1998). Reversible inactivation of protein-tyrosine phosphatase 1B in A431 cells stimulated with epidermal growth factor. J. Biol. Chem..

[83] Lee Y., Chang G. (2019). Quantitative display of the redox status of proteins with maleimide‐polyethylene glycol tagging. Electrophoresis.

[84] Wang S.-B., Foster D.B., Rucker J., O'Rourke B., Kass D.A., Eyk J.E.V. (2011). Redox regulation of mitochondrial ATP synthase. Circ. Res..

[85] Cobley J.N., Noble A., Jimenez-Fernandez E., Moya M.-T.V., Guille M., Husi H. (2019). Catalyst-free Click PEGylation reveals substantial mitochondrial ATP synthase sub-unit alpha oxidation before and after fertilisation. Redox Biol..

[86] Cobley J., Noble A., Bessell R., Guille M., Husi H. (2020). Reversible thiol oxidation inhibits the mitochondrial ATP synthase in Xenopus laevis oocytes. Antioxidants.

[87] Tuncay A., Crabtree D.R., Muggeridge D.J., Husi H., Cobley J.N. (2023). Performance benchmarking microplate-immunoassays for quantifying target-specific cysteine oxidation reveals their potential for understanding redox-regulation and oxidative stress. Free Radical Bio Med.

[88] Ferrer-Sueta G., Manta B., Botti H., Radi R., Trujillo M., Denicola A. (2011). Factors affecting protein thiol reactivity and specificity in peroxide reduction. Chem. Res. Toxicol..

[89] Lennicke C., Cochemé H.M. (2021). Redox metabolism: ROS as specific molecular regulators of cell signaling and function. Mol Cell.

[90] Choudhary D., Foster K.R., Uphoff S. (2023). Chaos in a bacterial stress response. Curr. Biol..

[91] Gleick J. (2008).

[92] Shannon C.E. (1948). A mathematical theory of communication. Bell Syst. Tech. J..

[93] Lloyd D., Aon M.A., Cortassa S. (2001). Why homeodynamics, not homeostasis?. Sci. World J..

[94] Cobley J.N., Fiorello M.L., Bailey D.M. (2018). 13 reasons why the brain is susceptible to oxidative stress. Redox Biol..

[95] Cobley J.N. (2018). Synapse pruning: mitochondrial ROS with their hands on the shears. Bioessays.

[96] Sidlauskaite E., Gibson J.W., Megson I.L., Whitfield P.D., Tovmasyan A., Batinic-Haberle I., Murphy M.P., Moult P.R., Cobley J.N. (2018). Mitochondrial ROS cause motor deficits induced by synaptic inactivity: implications for synapse pruning. Redox Biol..

[97] Nunn A.V.W., Guy G.W., Bell J.D. (2016). The quantum mitochondrion and optimal health. Biochem. Soc. Trans..

[98] Huang X., Chen S., Li W., Tang L., Zhang Y., Yang N., Zou Y., Zhai X., Xiao N., Liu W., Li P., Xu C. (2021). ROS regulated reversible protein phase separation synchronizes plant flowering. Nat. Chem. Biol..

[99] Kritsiligkou P., Bosch K., Shen T.K., Meurer M., Knop M., Dick T.P. (2023). Proteome-wide tagging with an H 2 O 2 biosensor reveals highly localized and dynamic redox microenvironments. Proc. Natl. Acad. Sci..

[100] Held J.M. (2020). Redox systems biology: harnessing the sentinels of the cysteine redoxome. Antioxid Redox Sign.

[101] Woo H.A., Yim S.H., Shin D.H., Kang D., Yu D.-Y., Rhee S.G. (2010). Inactivation of peroxiredoxin I by phosphorylation allows localized H2O2 accumulation for cell signaling. Cell.

[102] Sobotta M.C., Liou W., Stöcker S., Talwar D., Oehler M., Ruppert T., Scharf A.N.D., Dick T.P. (2015). Peroxiredoxin-2 and STAT3 form a redox relay for H2O2 signaling. Nat. Chem. Biol..

[103] Winterbourn C.C., Peskin A.V., Kleffmann T., Radi R., Pace P.E. (2023). Carbon dioxide/bicarbonate is required for sensitive inactivation of mammalian glyceraldehyde-3-phosphate dehydrogenase by hydrogen peroxide. Proc National Acad Sci.

[104] Lin S., Yang X., Jia S., Weeks A.M., Hornsby M., Lee P.S., Nichiporuk R.V., Iavarone A.T., Wells J.A., Toste F.D., Chang C.J. (2017). Redox-based reagents for chemoselective methionine bioconjugation. Science.

[105] Burnum-Johnson K.E., Conrads T.P., Drake R.R., Herr A.E., Iyengar R., Kelly R.T., Lundberg E., MacCoss M.J., Naba A., Nolan G.P., Pevzner P.A., Rodland K.D., Sechi S., Slavov N., Spraggins J.M., Eyk J.E.V., Vidal M., Vogel C., Walt D.R., Kelleher N.L. (2022). New views of old proteins: clarifying the enigmatic proteome. Mol. Cell. Proteomics.

[106] Davies M.J. (2016). Protein oxidation and peroxidation. Biochem. J..

[107] Desai H., Andrews K.H., Bergersen K.V., Ofori S., Yu F., Shikwana F., Arbing M.A., Boatner L.M., Villanueva M., Ung N., Reed E.F., Nesvizhskii A.I., Backus K.M. (2024). Chemoproteogenomic stratification of the missense variant cysteinome. Nat. Commun..

[108] Yoshikawa H., Larance M., Harney D.J., Sundaramoorthy R., Ly T., Owen-Hughes T., Lamond A.I. (2018). Efficient analysis of mammalian polysomes in cells and tissues using Ribo Mega-SEC. Elife.

[109] Winterbourn C.C. (2015). Are free radicals involved in thiol-based redox signaling?. Free Radical Bio Med.

[110] Winterbourn C.C. (1993). Superoxide as an intracellular radical sink. Free Radical Bio Med.

[111] Winterbourn C.C., Metodiewa D. (1994). The reaction of superoxide with reduced glutathione. Arch. Biochem. Biophys..

[112] J.N. Cobley, P.N. Chatzinikolaou, C.A. Schmidt, Computational Analysis of Human Cysteine Redox Proteoforms Reveals Novel Insights, (n.d.). 10.1101/2024.09.18.613618.

